# *Salvia rosmarinus* Callus as a Sustainable Source of Rosmarinic Acid and Its Anti-Inflammatory Potential and Toxicological Profile

**DOI:** 10.3390/biology15212041

**Published:** 2026-11-08

**Authors:** Aurelio Nieto-Trujillo, Alicia Monserrat Vazquez-Marquez, Nancy Aline Hernández-Rosas, Juan Luis Monribot-Villanueva, Angel Sahid Aguilar-Colorado, José Antonio Guerrero-Analco, Gabriel Alfonso Gutiérrez-Rebolledo, Porfirio Alonso Ruiz-Hurtado, Karla Mariela Hernández-Sánchez

**Affiliations:** 1 Centro de Investigación en Recursos Bióticos, Facultad de Ciencias, Universidad Autónoma del Estado de México, Carretera Toluca-Ixtlahuaca km 14.5, San Cayetano, Toluca 50295, Estado de México, Mexico; anietot_ext@uaemex.mx (A.N.-T.); alicia.vazmar@yahoo.com (A.M.V.-M.); 2 Laboratorio de Toxicología Molecular, Departamento de Farmacia, Escuela Nacional de Ciencias Biológicas, Instituto Politécnico Nacional, Alcaldía Gustavo A. Madero, Mexico City 07738, Mexico; alinehernandezr@hotmail.com; 3 Red de Estudios Moleculares Avanzados, Instituto de Ecología, A.C., Carretera Antigua a Coatepec 351, El Haya, Xalapa 91073, Veracruz, Mexico; juan.monribot@inecol.mx (J.L.M.-V.); angel.aguilar@inecol.mx (A.S.A.-C.); joseantonio.guerrero@inecol.mx (J.A.G.-A.); 4 Laboratorio de Toxicología de Productos Naturales, Departamento de Farmacia, Escuela Nacional de Ciencias Biológicas, Instituto Politécnico Nacional, Alcaldía Gustavo A. Madero, Mexico City 07738, Mexico; ggutierrezr@ipn.mx; 5 Laboratorio de Química de Productos Naturales, Departamento de Química Orgánica, Escuela Nacional de Ciencias Biológicas, Instituto Politécnico Nacional, Alcaldía Miguel Hidalgo, Mexico City 11340, Mexico

**Keywords:** plant biotechnology, *Rosmarinus officinalis*, cytotoxicity, acute toxicity, acute inflammation

## Abstract

In vitro plant culture is a biotechnological strategy that allows the conservation of biodiversity and the enhancement of pharmacological effects derived from secondary metabolite content in plants used traditionally in Mexican herbalism, such as *Salvia rosmarinus*. This study aimed to compare the phenolic profiles of two ethanolic extracts of *S. rosmarinus* from the leaves of plants and from the biomass of an in vitro callus culture, as well as their in vitro cytotoxicity, in vivo acute toxicity, and acute systemic and dermal anti-inflammatory effects. The results revealed that compared with the wild-type plant extract, the *S. rosmarinus* callus culture resulted in higher concentrations of three major metabolites, namely, rosmarinic acid, and in the in vitro cytotoxicity assay, only the callus line extract resulted in the same moderate activity (60% cell viability) against HeLa cells at all the tested concentrations (0.0039–0.250 mg/mL), whereas the activity of the wild-type plant extract was the same at higher concentrations (0.5–1 mg/mL); both extracts resulted in an LD_50_ > 2 g/kg BW after a single intragastric administration in male mice, but only for the wild-type plant extract was incipient kidney and spleen increased relative organ weight recorded at both intermediate and higher doses, which was not associated with lethality; in contrast, the liver weight of the callus extract was slightly altered at the low and intermediate doses tested. Finally, compared with the wild-type plant leaf extract, the extract of the callus biomass showed notable anti-inflammatory effects after single oral administration at the lower tested dose of 150 mg/kg, but with a weaker effect after single dermal application; however, it generated a moderate inhibition of the nitro-derived products in inflamed tissue concentration. The in vitro cell line of *Salvia rosmarinus* proved to be a viable, sustained and sustainable source of bioactive secondary metabolites such as rosmarinic acid, which are present at higher concentrations than those of wild-type plants.

## Simple Summary

In recent years, biotechnology-based plant cultures have been among the most important tools for both scientific development and various economic sectors (e.g., medical, pharmaceutical, and industrial), since the use of bioactive molecules from wild medicinal plants is always limited and coupled with increased environmental pollution, indicating that products derived from these plants can now show toxicity that has not been reported before, which is another advantage for their sterility and controlled conditions. In this study, a callus culture of *S. rosmarinus* was proven to be a source of rosmarinic acid, a phenolic metabolite proven to have several pharmacological effects, including anti-inflammatory effects, which also led to an increase in its biological effect when taken orally, similar to a well-known drug (e.g., indomethacin), and even greater in effect than the wild plant at lower doses; thus, it could be a source of biomass for the production of possible future phytomedicines focused on treating acute inflammation.

## 1. Introduction

Plants, which are among the medicinal species Salvia rosmarinus Spenn, have been essential sources of bioactive molecules for human health for millennia, mainly secondary metabolites with broad therapeutic potential, leading to the development of natural products for drug discovery and phytomedicine [1,2] (synonym of *Rosmarinus officinalis* L.), a perennial herb from the Lamiaceae family that is native to the Mediterranean region and is currently cultivated worldwide [3]. *Salvia rosmarinus* is recognized as an ornamental, medicinal and aromatic plant widely used as a fresh or dried herb in cooking recipes, herbal remedies, and personal hygiene and cosmetic products [4,5]. The pharmacological potential of *S. rosmarinus* is linked to its content of phenolic acids (mostly rosmarinic acid), flavonoids (such as diosmetin, hesperidin, apigenin, luteolin, cirsimaritin, genkwanin, homoplantaginin, and scutellarein), phenolic diterpenes (such as carnosic acid, carnosol, rosmadial, and rosmanol), monoterpenes (such as cineole, camphor, borneol, and α-pinene) and triterpenes (such as oleanolic, betulinic and ursolic acids) [6,7], which are related to its main reported pharmacological activities, including analgesic, antimicrobial, immunomodulatory, antioxidant and anti-inflammatory activities [3,7], with these last two compounds being interlinked through oxidative stress and inflammation respiratory bursts where free radicals such as reactive oxygen species (ROS) can directly activate inflammatory signaling pathways and immune cells, contributing to the development and progression of various chronic inflammatory diseases [6].

However, one of its major limitations is the low concentration of these secondary metabolites in normal basal wild plant tissues, resulting in a direct proportional relationship between the biochemical and metabolic adaptive processes of plants at different life stages and in response to adverse environmental conditions, which promotes an increase in the biosynthesis of polyphenolic acid derivatives [8,9,10].

Previous studies in different species of the *Salvia* genus, such as *Salvia nemorosa*, have shown how, through the cultivation of calluses from the roots of wild plants, it has been possible to establish a more sustainable culture of rosmarinic acid in higher proportions than in wild species [11], providing a great opportunity to establish an in vitro culture of calluses producing rosmarinic acid from a species found in Mexico and used in traditional herbal medicine, such as *Salvia rosmarinus*.

Plant tissue culture has become the most valuable method for the sustainable production of secondary metabolites; it consists of aseptic in vitro cultivation of cells, tissues, organs, or whole plants under controlled and defined conditions with respect to nutrient media and plant growth regulators. Specifically, a plant callus is a mass of proliferating cells that are not organized as a tissue and exhibit characteristics of parenchymatous cells and are formed from explants, which develop because of a process known as dedifferentiation or redifferentiation [12,13]. In vitro cultures present several advantages over wild field-grown plants: they are not subject to seasonal or climate variability, the need to harvest (many wild plants are endangered or slow-growing) is eliminated, and they allow the controlled and standardized production of selected secondary metabolites; moreover, they are also a viable solution in the face of water and soil pollution from toxins such as heavy metals that bioaccumulate in vacuoles, replacing the magnesium in chlorophyll, which gives previously harmless plants a toxic feature when ingested [8].

Plant metabolomics is dedicated to comprehensively exploring the intricate array of small molecules in plant systems. Targeted metabolomics involves the precise quantification and identification of specific metabolites by analyzing complex mixtures found within samples, employing sophisticated techniques because of their high selectivity and sensitivity, and offering an enhanced and more accessible path for drug discovery than traditional methods [14].

Owing to this context, the aim and innovation of this work were to compare the phenolic compound profile of ethanol extract from wild *S. rosmarinus* leaves to that of the biomass obtained from in vitro callus culture of the same species to compare their metabolomic profiles and evaluate their effects on edema formation, both intragastrically and systemically and through a dermal/local approach, as well as their effect on the tissue concentration of nitroderivative products during acute inflammation murine models.

## 2. Materials and Methods

### 2.1. Establishment of a Callus Line

A young *S. rosmarinus* plant was purchased from the commercial greenhouse “Viveros Martínez” in Calle Libertad 6 Calixtlahuaca, Toluca 50280, State of Mexico, Mexico, and served as the source of leaves for establishing a callus culture. Leaves were sequentially immersed for sterilization procedure previously described with some modifications [15]; in a solution of 1 g of powdered detergent (ROMA^®^, manufactured by 'La Corona' soap factory, Ecatepec de Morelos, Estado de Mexico, Mexico) for 15 min, an antibiotic solution 200 mg/L ampicillin (catalog number A5354, manufactured by Sigma-Aldrich, St. Louis, MO, USA) and 500 mg/L ciprofloxacin (catalog number 85721-33-1, manufactured by Sigma-Aldrich, St. Louis, MO, USA) for 30 min, a fungicide solution of 20 mL/L of Bravo^®^ 720 (COFEPRIS health registration number RSCO-FUNG-0309-430-008-054, manufactured by “Syngenta” factory, and distributed by “AMVAC Mexico S. de RL. De CV.”, Guadalajara, Mexico) for 30 min, a 70% ethanol solution (*v*/*v*) for 1 min, and a 20% chlorine solution (*v*/*v*, CLORALEX^®^, manufactured by the soap factory "AIEn" SA de CV, Monterrey, Nuevo León, Mexico) with three drops of Tween-20/L (catalog number 9005-64-5, manufactured by Sigma-Aldrich, St. Louis, MO, USA) for 20 min. Subsequently, under the laminar flow cabinet in sterile conditions, the leaves were rinsed 3 times with sterile distilled water, and three leaves were placed into each glass bottle in triplicate (n = 9).

The culture medium consisted of 50% Murashige and Skoog medium (using stock solutions prepared in the laboratory) supplemented with 2.5 mg/L of the auxin 2,4-dicholorophenoxyacetic acid (Sigma-Aldrich), 30 g/L sucrose (Sigma-Aldrich), 150 mg/L ascorbic acid (Sigma-Aldrich), 100 mg/L citric acid (Sigma-Aldrich), and 3 g/L phytagel (Sigma-Aldrich); the medium was adjusted to a pH of 5.7–5.8, and 25 mL was placed in each glass bottle and subsequently autoclaved at 121 °C for 18 min [16]. The resulting white and friable calli were maintained through subculturing every 30 days and harvested over a two-month period (December 2025 and January 2026); each time the calli were grown, a 1 g sample of the obtained biomass was taken and inoculated per flask (5 bottles in total), and they were kept in an incubation room under a 16 h photoperiod of indirect white, fluorescent light at a temperature of 27 °C ± 2, with the goal of keeping the callus culture viable and constant.

### 2.2. Preparation of Biological Material and Extraction

Leaves from a mature *S. rosmarinus* plant grown in a garden in Toluca, State of Mexico, Mexico, were collected in March 2026 and dried in an oven at 45 °C for 48 h. The species was taxonomically identified by comparison with specimens deposited in the herbarium of the Institute of Biology at the National Autonomous University of Mexico (UNAM, initials in Spanish) (voucher IBUNAM:MEXU:26365).

Callus tissue, obtained from two subsequent subcultures harvested in December 2025 and January 2026, and wild leaves were both dried under the same conditions (30 °C for 24 h), and then a mechanical grinding was done to get a fine powder in both dry samples. Taxonomic authentication of the in vitro callus line of *S. rosmarinus* was carried out by comparing the profile of polyphenolic components (4 taxonomic chemomarkers, mainly rosmarinic acid) with those of the ethanolic extract of the previously identified wild plant using the technique described in Section 2.3.

Subsequently, the extraction process was carried out in triplicate for dried wild leaves powder by the decoction technique (only one extraction batch was performed), which involved 10 g of dried powder; the same quantity was used from callus dried biomass (10 g), and it was put separately into 1 L Erlenmeyer flasks containing 500 mL of 75% ethanol and 25% distilled water, with the opening covered with aluminum foil and boiled for 30 min at approximately 75 °C. The solvent was then allowed to cool, and the biomass was removed via gravity filtration using Whatman filter paper(catalog number 1441-090, manufactured by GE Healthcare Life Sciences, Massachusetts, USA).

The extracts (*S. rosmarinus* wild leaf extract [*Sr*WE] and *S. rosmarinus* callus extract [*Sr*CE]) were evaporated in Petri dishes in an oven at 45 °C for 48 h; finally, the resulting solids were recovered by scraping the dishes with a spatula and stored in amber vials at room temperature.

### 2.3. Phenolic Compound Analysis of the Extracts by UPLC-MS

Phenolic compounds were identified and quantified by Ultrahigh-Performance Liquid Chromatography (UPLC) coupled with mass spectrometry (MS) as previously reported by Juárez-Trujillo et al. [17] and Monribot et al. [18], with some modifications according to the type of extract and vegetal species. Each sample was analyzed independently in triplicate, with three replicates of each above-described consecutive subculture.

#### 2.3.1. Sample Preparation and Chromatographic Conditions

The dried extracts were redissolved in methanol (50 mg/mL), filtered through 0.5 μm PTFE membranes, and placed in 2 mL UPLC vials with 0.3 mL inserts. Data were obtained with a 1290 Infinity Agilent UPLC system (Agilent Technologies, St. Clara, CA, USA) coupled to a 6460 Agilent triple quadrupole mass spectrometer (Agilent Technologies, St. Clara, CA, USA). The mobile phases were water with 0.1% formic acid (A) and acetonitrile with 0.1% formic acid (B), both in mass spectrometry (MS) grade, and the gradient conditions for the UPLC mobile phase are described in Table 1 as follows (total run time 45 min):

#### 2.3.2. Dynamic Multiple Reaction Monitoring (dMRM) by Mass Spectrometry (MS) Analysis

The MS was operated in negative and positive ionization modes using the parameters shown in Table 2. Phenolic compounds were identified using the dMRM method, with the precursor and product ions used as qualifier ions and the area of the product ion used for quantification (Table 3).

Calibration curves were constructed for each compound, which ranged from 0.125 to 19 µM, and the compounds were injected for three consecutive days. Quadratic regressions were used to obtain determination coefficients higher than 0.99 (Table 3). Data acquisition and processing were performed with MassHunter version B.06.00. The analytical method was validated by determining the quantification range (Table 3), where the concentration points allowed us to obtain determination coefficients higher than 0.99. Serial dilutions were made when the concentration of phenolic compounds was greater than 19 µM. The limit of detection was not evaluated, but it was declared when the concentration value is below the limit of quantification but is at least five times greater than the signal noise. The robustness of the analytical method was determined by standard injections during three consecutive days, resulting in a residual percentage lower than 20%. The precision and accuracy of the quantification method were determined by standard injections on the same day, resulting in a percent residual lower than 20%. Recovery and matrix-effect assessments were not evaluated.

### 2.4. In Vitro Toxicity

#### 2.4.1. Cell Culture

The cytotoxicity of the extracts of *S. rosmarinus* callus and wild plant leaves was analyzed in cervical cancer cells (HeLa ATCC identification code CCL-2) and noncancer immortalized human fibroblast (CHON-002 ATCC identification code CRL-2847) cell lines obtained from the Molecular Toxicology Laboratory of the National School of Biological Science, National Polytechnic Institute (ENCB-IPN initials in Spanish), Mexico. They were kept in culture media in accordance with the parameters established by the American Type Culture Collection (ATCC, Virginia, USA.), and both cell lines were cultured in Dulbecco’s Minimum Essential Medium (DMEM, catalog number 12100-046, manufactured by GIBCO, Grand Island, NY, USA) supplemented with 10% heat-inactivated fetal bovine serum (FBS, catalog number 16000-044, manufactured by GIBCO, Grand Island, NY, USA) and 1X penicillin/streptomycin/amphotericin B (catalog number ABL02, manufactured by CAISSON Labs, Smithfield, Utah, USA) (0.85% NaCl solution) in an incubator at 37 °C with a humidified atmosphere (5% CO_2_ and 95% air).

#### 2.4.2. Cell Viability Assay

The viability of *S. rosmarinus*-treated HeLa and CHON-002 cells was determined through a 3-(4,5-dimethylthiazol-2-yl)-2,5-diphenyl-tetrazolium bromide (MTT, catalog number M2003, manufactured by Sigma-Aldrich, St. Louis, MO, USA) assay at 1 mg/mL in fresh DMEM. HeLa and CHON-002 cells in exponential growth were seeded in 96-well plates at 2 × 10^5^ cells/mL and incubated for 24 h to stimulate their attachment. Different concentrations of both *S. rosmarinus* samples were applied to both cell lines and incubated for 24 h. The initial concentrations of both *Sr*WE and *Sr*CE were prepared at 1 mg/mL directly in DMEM media, due to the solubility of both freeze-dried extracts; therefore, non-solvent agents were used to test both extracts. The initial concentration tested in this work was determined according to the solubility determination of test substances in Annex 5 of the Organization for Economic Cooperation and Development (OECD) Guidance Document 129 [19], after which eight 1:2 dilutions were applied to HeLa and CHON-002 cells. Untreated cells and Paclitaxel (catalog number T7402, manufactured by Sigma-Aldrich, St. Louis, MO, USA) (assessed from 30 to 0.11 nM) were used as negative and positive controls (Absorbance of the X control), respectively; additionally, the absorbance at 540 nm of wells with the same extract concentrations tested without cells as interference controls in both cell lines; nevertheless, since the absorbances in these wells were similar to the wells with PBS and without cells, we concluded that the interference of both extracts was minimal; therefore, the absorbances of MTT in both cell lines were directly related to the effect of both extracts (Absorbance of the sample). The media of the control and treated cells were removed, and all the wells were washed twice with 100 µL of PBS. One hundred microliters of MTT reagent were added to both control and treated/exposed cells, and all of them were incubated at 37 °C for 4 h. After that, the MTT reagent solution was removed from the microplates, and the formazan salts in each well of the microplates were solubilized with 100 µL of dimethyl sulfoxide (DMSO, catalog number 1210, manufactured by MEYER, Mexico City, Mexico). The absorbance values at 540 nm were measured using an enzyme-linked immunosorbent assay (ELISA) reader (Bio-Tek model ELx800TM, manufactured by Aligent, Vermont, USA)) and compared with those of the untreated control cells in both cell lines. On the basis of the inhibition results, nonlinear regression tests were applied, and the median inhibitory concentration (IC_50_) was calculated. The percentage of cell viability was calculated with the following equation:

$$\%\;\mathrm{cell}\;\mathrm{viability}\;=\;100\;\times (\frac{\mathrm{Absorbance}\;\mathrm{of}\;\mathrm{the}\;\mathrm{sample}}{\mathrm{Absorbance}\;\mathrm{of}\;\mathrm{the}\;X\;\mathrm{control}})$$

Inhibitory concentration (IC_50_) values were determined by non-linear regression analysis using a four-parameter logistic (4PL) dose–response curve in GrpahPad Prism 10.2.0. Data were normalized as percentage inhibition relative to vehicle-treated controls, and inhibitor concentrations were plotted on a log_10_ scale. Results are expressed as the IC_50_, 95% of confidence intervals (95% CI) in mg/mL from *n* = 3 independent experiments. Goodness of fit was verified by R^2^ > 0.95 for all curves.

### 2.5. In Vivo Models

#### 2.5.1. Laboratory Animal Storage and Health Conditions

The total population (N_T_) of 150 CD1-strain healthy first-use male mice (25 ± 5 g of body weight [BW], 6 weeks old) was maintained in an acclimatization period for one week under controlled vivarium conditions: inverted light/dark cycles of 12 h/12 h, humidity of 60–80%, temperature of 22 ± 5 °C, and access to purified water and food (RodentChow 5001) before the experiments began.

The total population was randomly distributed across the various acute murine models as follows: 24 for acute toxicity, 56 for carrageenan-induced paw edema, and 70 for ear edema induced by 12-O-tetradecanoyl phorbol-13-acetate (TPA). In each in vivo model, the distribution of animals per experimental group, dosages, administration volumes and the vehicle in which both extracts were diluted are described in their specific section.

All procedures described in this work were performed following guidelines described in Mexican Official Normal NOM-062-ZOO-1999 [20] modified in 2001, revised in 2026, and were reviewed and endorsed by the Institutional Committee for the Care and Use of Laboratory Animals (CICUAL) and Ethics Committee of the Autonomous University of the State of Mexico (UAEMex), Toluca, Mexico.

#### 2.5.2. Acute Toxicity Model

The median lethal dose (LD_50_) was established following the guidelines described in Test Guideline (TG) 423 of the OECD [21]. Twenty-four male mice were randomly distributed (n = 6) in two independent and serial experiments in a 1:1 ratio using a pseudo-random number generator on a scientific calculator as follows: (1) vehicle group, (2) 2000 mg/kg BW extract group, (3) 200 mg/kg BW group, and (4) 20 mg/kg BW group. All healthy animals were fasted for 6 h prior to single intragastric (i.g.) administration. The control group (1) was administered only the vehicle (tween 80: purified water, proportion 1:9, catalog number P1754, manufactured by Sigma-Aldrich, St. Louis, MO, USA) at 10 mL/kg BW, and all the vehicle-resuspended treatments were administered by the same i.g. route at 10 mL/kg BW; to ensure concealment, the allocation sequence was generated by a researcher who was not involved in the subsequent experimental procedures. The outcome assessment was fully blinded. The researchers who evaluated the preclinical outcomes and analyzed the data were completely unaware of the experimental mouse group assignments throughout the study.

Six hours after a single and unique administration of each treatment (20, 200 or 2000 mg/kg doses of both extracts in each experimental mouse group at the beginning of the study), any behavioral alterations (sedation, hypnosis, convulsions, aggression, lethargy, hyperactivity and piloerection) were recorded every day until two weeks, at which time the body weight (BW) was also measured on days 1, 3, 7, 9 and 14. Any death that occurred was recorded, and a rigorous necropsy was performed for macroscopic evaluation of major organs. To correct for individual baseline variations, BW data were normalized as expressed as percentage change relative of each measurement day (BW at the Day X) comapared to Day 1 (the start of treatment, BW at the Day 1) in accordance with the experimental design criteria recommended by the ARRIVE guidelines [22]. The percentage change in BW was calculated with the following formula:

$$\mathrm{Normalized}\;\mathrm{BW}\;\left(\%\right)=\frac{\mathrm{BW}\;\mathrm{at}\;\mathrm{the}\;\mathrm{Day}\;X}{\mathrm{BW}\;\mathrm{at}\;\mathrm{the}\;\mathrm{Day}\;1}\;\times \;100$$

Statistical comparisons were performed using a two-way repeated-measures ANOVA and Tukey’s post hoc test analyzed by GraphPad Prism software version 10.2.0, with the weight on the Day 1 included as a covariate to maximize statistical power and reduce bias stemming from initial body mass [23].

On day 14, the surviving animals were euthanized by cervical dislocation to perform corresponding necropsies, as well as main organ extraction, for macroscopic evaluation (coloration and general appearance), and the relative weight of each was subsequently calculated with the following formula:

∆ *Organ weight* = *Organ weight at Day X* − *Organ weight at Day* 1

#### 2.5.3. Acute Paw Edema Induced by λ-Carrageenan

In accordance with the methods of Gutiérrez-Rebolledo et al. [24], 56 male mice were randomly distributed into each experimental group (n = 7): (I) control λ-carrageenan (catalog number 22049, manufactured by Sigma-Aldrich, St. Louis, MO, USA) without treatment and (II) the reference drugs indomethacin (catalog number PHR1247, manufactured by Sigma-Aldrich, St. Louis, MO, USA) (10 mg/kg BW), (III–V) *Sr*WE, and (VI–VIII) *Sr*CE, each at doses of 150, 300, and 600 mg/kg BW. The doses proposed for evaluation were based on previous studies in male Wistar rats in which a median effective dose (ED_50_) of 300 mg/kg by i.g., single administration was calculated for the acute subplantar edema model induced by carrageenan [25].

All treatments were reconstituted in the vehicle (Tween 80:water, 1:9, catalog number P1754, manufactured by Sigma-Aldrich, St. Louis, MO, USA) and administered once i.g. at 10 mL/kg BW one hour before subcutaneous (s.c.) injection of 20 µL of carrageenan (2%, in sterile isotonic saline solution, ISS) into the hind paw cushion.

Measurements were performed using a digital Vernier (Mitutoyo model 293–831, manufactured by Mitutoyo, Utsunomiya City, Japan), which was used to measure the time zero (Eo) basal diameter of the paw cushion (mm) before the injection of the phlogistic agent s.c., and a measurement was taken at 5 h (Et) (maximum formation of edema). The percentage of inhibition was calculated using the following formula:

$$\%\;\mathrm{inhibition}=[\frac{\left(\mathrm{Et}-\mathrm{Eo}\;\mathrm{control}\;\mathrm{carrageenan}\right)-\left(\mathrm{Et}-\mathrm{Eo}\;\mathrm{treated}\;\mathrm{group}\right)}{\left(\mathrm{Et}-\mathrm{Eo}\;\mathrm{control}\;\mathrm{carrageenan}\right)}\;\times \;100]$$

#### 2.5.4. Acute Ear Edema Induced by 12-O-Tetradecanoylphorbol-13-Acetate (TPA)

In accordance with the methods of Gutiérrez-Rebolledo et al. [22], 70 male mice were randomly distributed into each experimental group (n = 7): (I) control TPA (Sigma-Aldrich P8139) without treatment, (II–IV) the reference drug indomethacin (Sigma-Aldrich PHR1247), (V–VII) *Sr*WE and (VIII–X) *Sr*CE. All treatments were tested at 0.5, 1, and 2 mg/ear. The amounts assessed are those established in previous studies on the basis of the solubility of the samples in the acetone solvent [24].

An amount of 25 µL of TPA (2.5 µg) dissolved in acetone (vehicle) was applied to the right ear (Ws), and only 25 µL of acetone was applied to the left ear (Wo). After 30 min, all treatments reconstituted in acetone (50 µL) were applied to the right ear (Ws).

Six hours after TPA was topically applied (edema maximum formation), all the mice were euthanized by cervical dislocation, and sections of central auricular tissue (6 mm) were extracted and weighed to calculate the amount of auricular edema formed (mg), and the percentage of inhibition (%) was determined with the following formula:

$$\%\;\mathrm{inhibition}=[\frac{\left(\mathrm{Ws}-\mathrm{Wo}\;\mathrm{control}\;\mathrm{TPA}\right)-\left(\mathrm{Ws}-\mathrm{Wo}\;\mathrm{treated}\;\mathrm{group}\right)}{\left(\mathrm{Ws}-\mathrm{Wo}\;\mathrm{control}\;\mathrm{TPA}\right)}\;\times \;100]$$

##### Quantification of Tissue Nitro-Derived Products

Measurement of nitrite (NO_2_^−^), a primary and nonvolatile product, was performed in the tissue samples through the Griess reaction [26,27]. This technique was applied only to quantify the tissue concentration of nitroderivative products, like NO•, but it was not considered a specific technique that can faithfully interpret, indirectly, the activity of iNOS.

After acute ear edema tissue (6 mm) from all the experimental groups was obtained and weighed, each one was homogenized in 1 mL of cold phosphate buffer solution (PBS, pH 4.3). Immediately after, the homogenates were centrifuged at 10,000 rpm for 5 min at 4 °C to isolate the supernatants; then, 50 µL of each supernatant was transferred to each empty well of a 96-well plate, mixed with 50 µL of sulfanilamide (catalog number S9251, manufactured by Sigma-Aldrich, St. Louis, MO, USA) (1% in 5% phosphoric acid–water solution), and incubated at room temperature (25 ± 5 °C) for 10 min in the dark.

Finally, 50 µL of N-1-naphtylethylenediamine dihydrochloride (NED, catalog number 222488, manufactured by Sigma-Aldrich, St. Louis, MO, USA) was added to each well and then incubated at room temperature (25 ± 5 °C) in the dark for another 10 min. The absorbance was measured at 540 nm in an ELISA microplate reader (model ELx800TM, manufactured by Aligent, Vermont, USA). To determine the quantity of NO_2_^−^ in each sample, a standard curve of NO_2_^−^ (catalog number 237213, manufactured by Sigma-Aldrich, St. Louis, MO, USA) from 100 µM to 0 µM was constructed in triplicate (n = 3) following the same protocol previously described (Y = 0.0035x + 0.0529; R^2^ = 0.9997).

The results were reported only as the approximate tissue concentration of nitro-derivative products present in the entire acute ear edema, as interpolated on the calibration curve, so they were reported just as the concentration of nitroderivative products [µM], without relating it to grams of tissue, since the maximum formation of ear edema in untreated animals was about 24 mg.

### 2.6. Statistical Analysis

Metabolite quantification results were analyzed using Welch’s *t*-tests in Metaboanalyst 6.0; the data were transformed by cube root and scaled; for the autoscaling, the data were mean-centered and divided by the standard deviation of each variable. In addition, Metaboanalyst transformed the zeros (Nondetected compounds) as half (1/2) or one-fifth (1/5) of the minimum positive value found in the original dataset to perform statistical analyses.

For the in vitro results of the cytotoxicity assay, the IC_50_ values were calculated as the concentrations that show 50% inhibition of proliferation in any tested cell line using the GraphPad Prism software version 10.2.0. statistical program, where nonlinear regression tests and calculations of the median inhibitory concentration were performed. Two-way ANOVA with repeated measures (RM) and post hoc Šídák multiple comparison test (*p* < 0.05) were carried out.

For the in vivo acute toxicity model, GraphPad Prism software version 10.2.0. was used. For each result, a Shapiro-Wilk normality test was performed (*p* > 0.05). For the results of the BW during the acute toxicity model, two-way ANOVA with repeated measures (RM) and post hoc Tukey tests (*p* < 0.05) were carried out, whereas for the relative weight of organs (%), Shapiro-Wilk tests and Brown–Forsythe tests were performed to confirm the homogeneity of variance; robust Welch’s ANOVA and post hoc Dunnett’s T3 multiple comparison tests (*p* < 0.05) and ordinary one-way ANOVA and Tukey’s multiple comparison test (*p* < 0.05) were carried out.

Finally, a simple two-way ANOVA RM was applied only for the carrageenan results, and for the TPA results, a two-way ANOVA was applied. In both cases, a post hoc Student-Newman-Keuls (SNK) test was performed, with a significance value of *p* < 0.05.

### 2.7. AI Tools

For the preparation of this study, the authors used FigureLabs artificial intelligence tools to generate the graphical abstract. The authors reviewed and edited the final image generated with this tool, whose certificate ID is FL-PUB-20260923-Y0JPTV, which authorizes the commercial use and publication of this figure; therefore, we assume responsibility for the content of this publication.

## 3. Results

### 3.1. Phytochemical Profile

The extraction conditions resulted in an extraction yield of 30.53 ± 4.25% for the leaves and 46.16 ± 7.92% for the calli, thereby yielding more extract from the in vitro culture.

The results of the UPLC-MS analysis of selected phenolics used as taxonomic chemomarkers revealed the presence of vanilloside and chlorogenic acid in the callus extract of *S. rosmarinus*; however, none of these compounds were detected in wild-type plants. There were no significant differences in the coumaric acid content between the two extracts, but the callus culture extract exerted higher concentrations of protocatechuic, ferulic, and rosmarinic acids than wild plant extract. Specifically, the callus culture extract contained 122% more rosmarinic acid than the leaf extract (Table 4).

### 3.2. Toxicological Profile

#### 3.2.1. Cell Viability Assays

**Figure 1:**
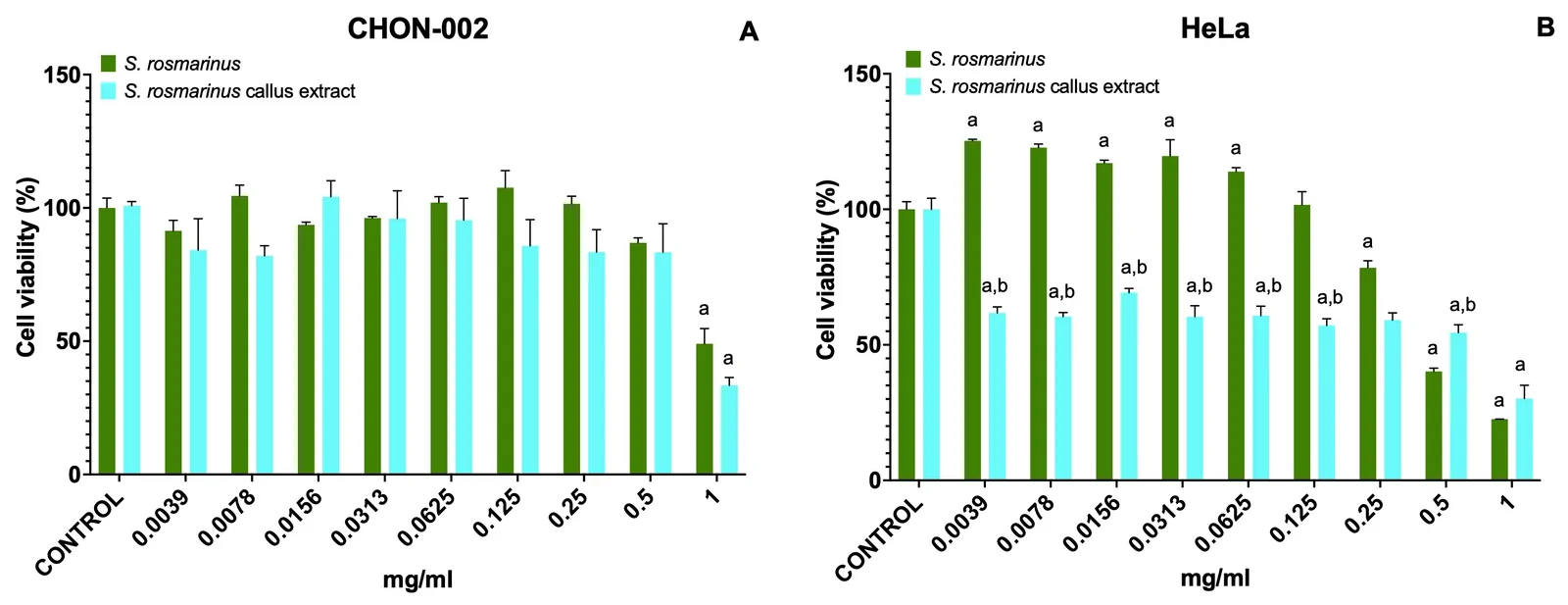
Effects of *S. rosmarinus* callus line and wild-type plant leaf extracts on (**A**) CHON-002 and (**B**) HeLa cell viability. The data are shown as the mean (±) and SEM (n = 3). Shapiro-Wilk normality tests, two-way ANOVA with repeated measures (RM), and post hoc Šídák multiple comparison test (*p* < 0.05) were carried out. Superscript letters (a) indicate significant differences vs. the corresponding control cell group; (b) indicate significant differences vs. the *S. rosmarinus* wild-type extract at its own concentration level.

In vitro analysis of wild-type *S. rosmarinus* extract revealed a cytotoxic effect on CHON-002 cells, with a 51% decrease in cell viability only at the higher concentration evaluated (1 mg/mL); in the same form, *S. rosmarinus* callus extract also had a cytotoxic effect, with a decrease in cell viability of 67% at only the higher concentration evaluated (Figure 1A). In contrast, wild-type *S. rosmarinus* extract reduces the viability of HeLa cells from 0.25 to 1 mg/mL; nevertheless, concentrations from 0.125 mg/mL increase cell viability to 25% for HeLa cells. On the other hand, *S. rosmarinus* cell extract reduced the viability of HeLa cells at all concentrations evaluated, with a reduction in cell viability of 40% at the lower concentration evaluated and a 70% decrease in cell inhibition at the higher concentration used (Figure 1B).

In this sense, owing to the concentration-dependent effect of the wild-type *S. rosmarinus* extract on HeLa cells, the IC_50_ (0.3262 mg/mL, 95% CI: 0.2367 to 0.3973 mg/mL) could be calculated; this result was higher than the IC_50_ of Paclitaxel (1.52 × 10^−5^ mg/mL, 95% CI: 4.883 × 10^−6^ to 1.588 × 10^−5^ mg/mL) in this cell line, which was similar to the IC_50_ reported for this cell line (15.7 ± 1.6 nM) in another study [28, 29]. On the other hand, the IC50 of callus extract cannot be calculated because the cytotoxic effect displayed on HeLa cells at the concentrations evaluated did not permit a minimal accurate curve fit to determine this value.

#### 3.2.2. In Vivo Acute Toxicity

In accordance with the acute toxicity test described in OECD 423, signs of animal welfare were recorded in all experimental groups throughout the experimental acute toxicity evaluation; in reference to the observation of animals at each evaluated dose in the first four to six hours after single administration, the animals were monitored to record any signs of toxicity, with special attention given to changes related to skin and fur, eyes and mucous membranes; changes in respiratory and neuronal alterations; and episodes of tremors, convulsions, excessive salivation, diarrhea, lethargy, sleep, and coma [21]. Any change was systematically recorded and contrasted with a control group to determine any toxicological alterations in animal welfare; nevertheless, in all cases, any of the previously mentioned toxicological alterations were present in the treated animals at any evaluated dose (Table 5 and Table 6).

**Figure 2:**
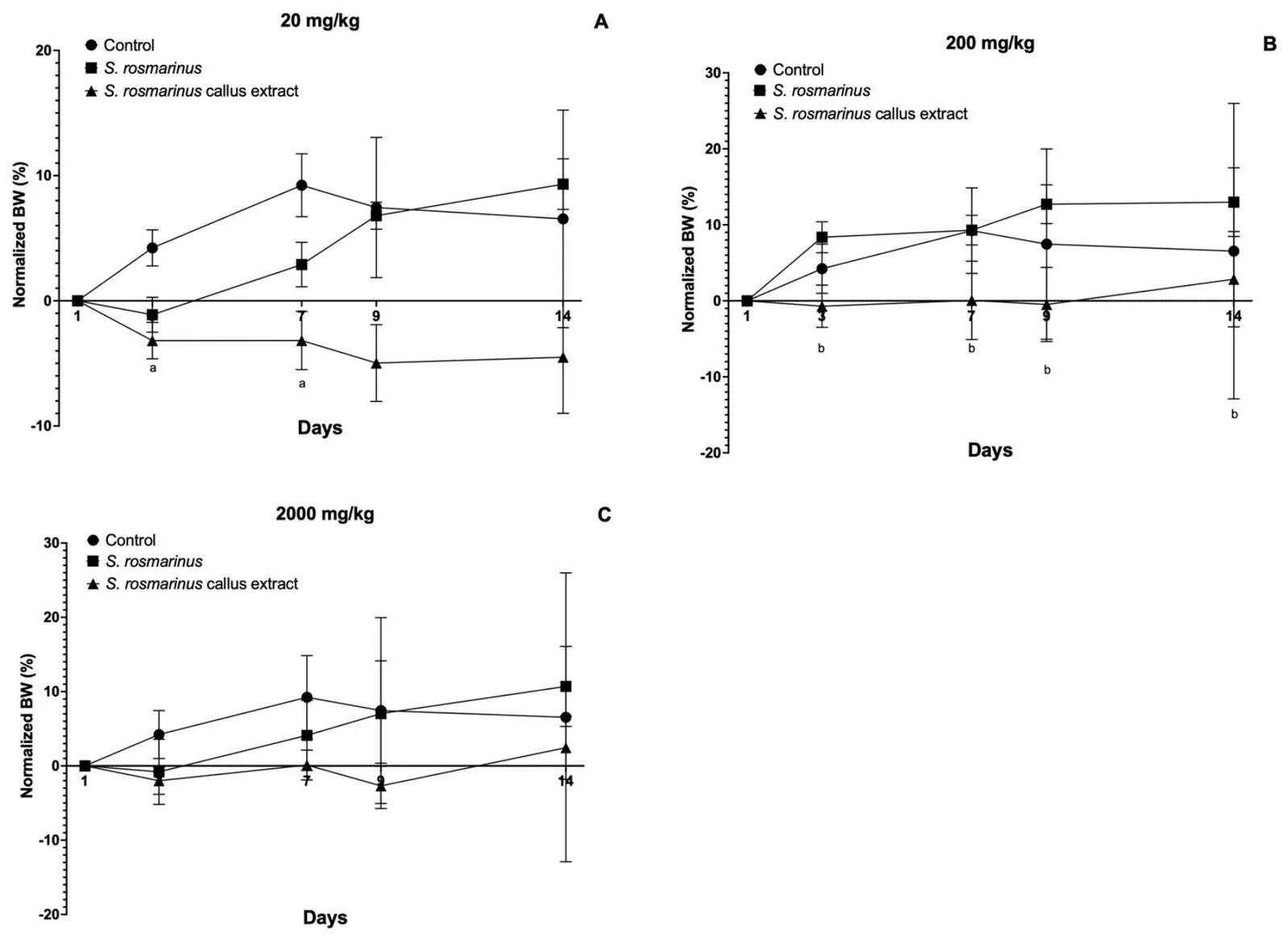
Effects of *S. rosmarinus* callus line and wild plant leaf extracts on body weight variation. The data are shown as the mean (±) and SEM (n = 6) of (**A**) 20 mg/kg doses; (**B**) 200 mg/kg doses; and (**C**) 2000 mg/kg doses. Treatments were administered once i.g.; the Shapiro-Wilk normality test, two-way ANOVA RM, and post hoc Tukey test (*p* < 0.05). Superscript letters (a) indicate significant differences vs. the control mouse group, and (b) indicate significant differences vs. the *S. rosmarinus* wild-type extract group at its own dosage level.

Moreover, the individual total weights of the animals were systematically recorded. The variation in body mass (%) of the animals treated with each of the tested extract doses is shown in Figure 2, in contrast with the control group. Notably, compared with the control group, the group treated with the *S. rosmarinus* callus line at 20 mg/kg presented a BW loss closer to 6% between the 3rd day and the 7th day post-treatment (Figure 2A); however, no death occurred in this experimental group. In contrast, higher doses of extract (200 and 2000 mg/kg BW) did not induce alterations in body weight compared with those in the control group (Figure 2B,C); lethality was not observed. On the other hand, the body weight of the wild-type *S. rosmarinus* extract-treated group did not significantly differ from that of the respective control group (Figure 2A–C).

Major pathological analysis was carried out after the animals were euthanized, and total changes in body weight were calculated through systematic recording of the individual body weights of each treated animal at the different tested doses, and the results revealed that the BW of the *S. rosmarinus* callus line extract decreased in all cases in a dose-dependent manner (Table 7). In comparison, *S. rosmarinus* wild-type extract at all doses did not influence the total BW compared with that of the control group (Table 7).

Compared with those in the control group, the relative weight of the liver in the mice treated with *S. rosmarinus* callus line extract at doses of 200 and 2000 mg/kg (0.06382 g and 0.07155 respectively) increased with respect to the control group (0.05927 g); in contrast, the weight of the liver in the mice treated with *S. rosmarinus* wild-type plant leaf extract at all doses did not increase compared with that in the control group (Table 7).

On the other hand, compared with those in the control group, the relative masses of the kidneys in the groups treated with *S. rosmarinus* extract at doses of 20 and 2000 mg/kg increased by approximately 20% and 30%, respectively, on the 14th day; notably, compared with those in the control group, *S. rosmarinus* at the higher dose (2000 mg/kg) significantly differed (Table 7).

Finally, compared with those of the control group, the relative spleen weights of the mice treated with only the *S. rosmarinus* extract at all doses significantly increased; in contrast, the weights of the mice treated with the *S. rosmarinus* callus line extract did not significantly differ from those of the control group at any tested dose (Table 7).

In accordance with the criteria and flow map of Annex 2d and 3 of the acute toxicity OECD 423 test method [21], and considering that the LD_50_ is probably >2000 mg/kg for both the extracts assayed by the i.g. route in male CD1 mice, together with the animal welfare parameters included in the toxicological protocol adopted and both the total body weight variation and the relative organ weight variations observed in the experimental groups, both the callus line and the wild-type *S. rosmarinus* extracts assessed in this work can be classified into category 5 of the Globally Harmonized System (GHS) [30]. Nevertheless, it is worth mentioning that biochemical parameters related to organs such as the liver, kidney, and spleen should be evaluated in subsequent studies of long-term administration to precisely assess the safety of both extracts, despite not having shown lethality, indicating that both extracts have a lower oral acute toxicity effect.

### 3.3. In Vivo Anti-Inflammatory Evaluation

#### 3.3.1. Systemic Effects

Single s.c. injection in animals in the untreated carrageenan control group caused, at the 5th hour, an edema diameter increased to 0.97 ± 0.02 mm (the maximum degree of inflammation before the natural resolution of inflammation occurred at 7 h after s.c. inoculation with the inflammatory agent), whereas compared with the reference drug control treatment, a single i.g. administration of indomethacin significantly inhibited paw edema (44.56%) at 10 mg/kg (Table 8). However, the anti-inflammatory effect of the extract from callus of *S. rosmarinus* showed no statistically significant difference from that of indomethacin when it was administered at doses of 300 and 600 mg/kg (approximately 44%); moreover, compared with the carrageenan untreated control group, the group administered with 150 mg/kg of callus extract had a significant anti-edema effect (37.12%), and its effect was significantly lower than that of the reference drug and the other two evaluated doses (Table 8).

On the other hand, the wild-type plant extract had a markedly dose-dependent antiedematous effect, since at 150 mg/kg, no effect was detected; at 300 mg/kg, a significant antiedematous effect was observed (27.61%) compared to carrageenan un-treated control group at same hour, although it was weaker than that of the reference drug and the callus line extract, however dose of 600 mg/kg exerted significant greater anti-inflammatory effect (58.10%), even than the reference drug (Table 8).

#### 3.3.2. Local Dermal Effect

Single direct application of the irritant agent TPA to the mouse ear skin resulted in the development of acute inflammation, with maximum ear edema formation of 23.90 ± 0.82 mg at 6 h after the irritant skin agent was applied; the results revealed that the *S. rosmarinus* callus extract did not show a quantity-dependent local anti-inflammatory effect, since at 1 and 2 mg/ear analysis showed that no statistically significant difference were found, but was significantly less potent than the reference drug at the same quantities (1 and 2 mg/ear), which inhibited auricular edema formation by 36.40 and 61.51%, respectively. However, at 0.5 mg/ear, the antiedematous effect of the callus extract was significantly similar to that of the reference drug at the same applied quantity (approximately 20%) (Table 9).

The local/dermal anti-inflammatory effect of the wild-type plant extract was similar to that of the callus line extract; it was not quantity dependent, since at 0.5 mg/ear, it significantly inhibited ear edema by 29.71%, with the similar effect as that of the other two tested samples; however, at 1 mg/ear, it had significantly greater antiedematous activity (41.84%) than that exerted by the callus extract (24.69%), but it was significantly greater than that of the reference drug (36.40%) both at the same amount, however it was significantly lower (38.91%) at 2 mg/ear compared to indomethacin at same tested amount (61.51%) (Table 9).

##### Tissue Concentration of Nitro-Derived Products in Acute Ear Edema

A single dermal application of TPA generated a higher nitro-derived products tissue concentration during acute ear edema of 15.25 ± 0.93 µM at 6 h post-application, related to an increase in these during acute inflammation, like NO• [23]. Since the action mechanism of indomethacin is not related to the generation of NO•, its three tested quantities significantly inhibited nitro-derived product generation in a similar proportion (approximately 27%), compared with the untreated TPA control (Table 9).

The extract from the *S. rosmarinus* callus line was able to inhibit the tissue nitro-derived products concentration in a quantity-dependent manner, with 0.5 mg/ear significantly inhibiting it by 86.16%, whereas the amounts of 1 and 2 mg/ear were significantly similar (94.5%), compared with the untreated TPA group (15.25 ± 0.93 nitro-derived products µM). Finally, wild plant extract showed a significant decrease in tissue nitro-derived products concentration, with a behavior similar to callus line extract, quantity-dependent, since at 2 mg/ear it showed an inhibition of 69.57%, while 1 and 0.5 mg/ear showed no statistically significant difference related to inhibitory response on nitro-derived products tissue concentration (approx. 58.5%), highlighting that wild plant extract therefore showed half the effect displayed by callus line extract, and also all tested quantities were significantly different from untreated TPA control, and showed greater inhibition than indomethacin at same quantities (Table 9).

## 4. Discussion

In this study, *Salvia rosmarinus* calluses displayed an important advantage with respect to their respective wild form because the high degree of variation in chemical composition is usually closely related to the geographical location of growth and harvest and their vegetative cycle [3,31]. Moreover, compared with wild plants, plants in in vitro culture systems are recognized by the scientific community because they offer advantages, such as the rapid, controlled, large-scale propagation of plants with pathogen-free and disease-free organisms that offer a constant production of therapeutic phytochemicals with a constant composition and content of bioactive secondary metabolites [32,33,34]. There is evidence that calluses enhance the biological properties of wild medicinal plants such as *Tephrosia tinctoria,* which increase their cytotoxic activity against the HepG2 cancer cell line, although other chemical properties of *T. tinctoria* callus culture, such as its antioxidant potential, are reduced [35].

These results of the cell viability assay are consistent with the cytotoxic effect reported in other works of wild-type *S. rosmarinus* ethanolic extract against the HeLa human cancer cell line from 0.3 to 0.5 mg/mL [36,37,38]. In contrast, the extract from the callus of *S. rosmarinus* displays a greater cytotoxic effect against HeLa cells, which begins at 0.0039 mg/mL, indicating that the cellular culture of *S. rosmarinus* increases its cytotoxicity in this specific cell line; this is important because our results, taken together with those of other authors, could provide the possibility to explore the anticancer effects of *S. Rosmarinus,* both wild and callus extract, in future works.

In terms of the anticancer potential of *S. rosmarinus* that has been studied in other works and that reinforces the potential effect of this plant, we found that an ethanolic extract from this wild-type plant inhibits the secretion of IL-6 in HeLa cells [38], which is a key interleukin involved in the promotion and proliferation of tumor processes and is related to the chemoresistance of tumors derived from Caco-2 (colorectal), HeLa (cervicouterine), MCF-7 and HT-29 (both from breast) cells [39,40]. This cytotoxic activity is also related to the anti-inflammatory potential of *S. rosmarinus* because of the phenolic compound content, such as rosmarinic acid, which has the capacity to inhibit the NF-kB pathway and is slightly involved in IL-6 transcription [41,42,43].

The greater cytotoxic effect of the *S. rosmarinus* callus extract on HeLa cells could be related to the higher concentration of bioactive compounds present in its chemical composition. Therefore, the increase of approximately 122% in this phenylpropanoid in the *S. rosmarinus* callus extract with respect to that in the *S. rosmarinus* wild-type plant extract is a key factor that can explain this point. Finally, the cytotoxicity of *S. rosmarinus* to HeLa proliferation is possibly closely related to its phenolic compound content, such as rosmarinic and ferulic acids, and its cellular antioxidant capacity to decrease the impact of ROS on cellular DNA and membranes and therefore reduce oxidative damage to cell membrane integrity in cancer cells, inducing cell death through mechanisms other than lysis [38].

On the other hand, this is the first study to evaluate the cytotoxic effects of both *S. rosmarinus* wild-type plant extract and callus extract on the CHON-002 (normal/healthy human articular chondrocyte) cell line. In both extracts assayed, only a significant reduction of approximately 50% in cell viability was determined at the highest concentration evaluated; however, the next concentration evaluated in both extracts (0.5 mg/mL) remained close to 85% of the cell viability, which is consistent with the value reported by other authors in nonmalignant cells, such as the human mammary epithelial cell line MCF-10A (breast cancer), in which the concentration of 0.4 mg/mL *S. rosmarinus* ethanolic extract and essential oil extract remained above 80% of the cell viability [44].

*S. rosmarinus* is usually considered a safer medicinal plant that can be used for both consumption and dermatological approaches; nevertheless, herbal medications and formulations from this medicinal plant can have adverse side effects in patients [45,46]. In this work, although both *S. rosmarinus* callus and wild plant leaf ethanolic extracts were classified into the five categories of GSH, the classification method used in this work was based on observed mortality criteria to classify the lethal potential [30].

Some alterations in body and organ relative weights were recorded; these findings contradict those reported by other authors regarding the safety and no alterations observed with the higher tested dose (2000 mg/kg) by i.g., single administration of wild-type *S. rosmarinus* leaf extract in Wistar rats [47].

The authors reported no abnormal signs, behavioral changes, body weight, or food and water alterations related to the dosage of wild *S. rosmarinus* leaf extract during the two weeks of animal monitoring, which also supported the lower toxicity of hydroalcoholic extract of rosemary in a repeated-dose i.g. administration for 28 days in a rat model [48]. Nevertheless, it is important to highlight that the animal model used in this work differs; on the one hand, the toxicological profiles of both CD1 mice and Wistar rats can differ, and there is evidence that, compared with Wistar rats, CD1 mice show greater marked hepatotoxicity, which can be explained by their differences in metabolic rates and intrinsic clearance values [49].

An important consideration in the toxicological evaluation of *S. rosmarinus* formulation-based products is the differences in their chemical composition [45]; this is supported by a study conducted with an oil-soluble rosemary extract in which this formulation, although it did not show mutagenic effects through an evaluation with the Ames assay, was continuously administered in the diet of female experimental rats of this rosemary extract for 90 days; after this time period, liver enlargement was completely reversible after 28 days; additionally, the reversible stimulation of some cytochrome P_450_ enzymes indicated that the enlargement of the liver was an adaptive response and was not a toxicological consequence of the treatment with the rosemary extract [50]. This evidence could explain the relative weight increase in the liver observed in this work for mice treated with *S. rosmarinus* callus and wild-type extract, which was not associated with lethality.

However, this study has several limitations, such as the absence of histopathological and biochemical analyses related to liver, kidney, and spleen toxicological evaluation; therefore, although a significant increase in the relative liver weight was shown in this work in response to treatment with the callus extract, this does not reflect an obvious hepatotoxic effect of *S. rosmarinus*, and further toxicological studies involving repeated administrations are needed. Another limitation is that although phenolic compounds are expressed in µg per gram of extract, this value does not necessarily imply higher or lower concentrations per gram of vegetal tissue. One of the limitations of this study is that its anti-inflammatory effect was not tested in female animals and that there was a lack of quantification of certain inflammatory biomarkers.

Notably, compared with the control treatment, the wild-type rosemary extract used in this work also increased the kidney and spleen relative weights, which was not observed in any of the rosemary callus extract treatments. This finding is in agreement with the findings of other studies conducted with Wistar rats treated with rosemary essential oil, which induces degenerative and trophic alterations in the kidney and liver, increases the levels of blood urea nitrogen and AST enzymes, and affects hepatocytes central to the distal vein diameter [51]. Therefore, it is important to carry out more studies on the renal and spleen effects of *S. rosmarinus* wild extracts at different dosages and longer treatment periods; in this sense, compared with the wild-type rosemary extract, the rosemary callus extract could be advantageous because it did not exhibit observable alterations in either the kidney or the spleen at any dosage evaluated.

The acute subplantar edema model induced by a single s.c. injection of carrageenan is widely used to evaluate the effectiveness of potential new molecules or water-soluble agents that are easily absorbed in the gastrointestinal tract to act systemically as COX-2 inhibitors when orally administered, such as phenolic acids and flavonoids, which is why the reference drug used is nonsteroidal anti-inflammatory drugs (NSAIDs), such as indomethacin, during the 7 h experimental acute inflammatory process [52]; however, it is worth highlighting the behavior of both extracts at hour 3 of the model, since it is at this early stage, when vasodilation is activated, that their antiedematous effect because the secondary metabolites present in both extracts of *S. rosmarinus* could be capable of regulating vasodilatory activity typical of acute inflammation, as described in this model from 0 to 4 h after carrageenan s.c. injection, by inhibiting iNOS activity [53].

In this context, TPA is a skin proinflammatory agent used to evaluate possible COX-2 inhibitors absorbed by the dermis, with two well-known and well-studied phases of acute ear edema: (1) from baseline (time zero) to 3 h after the application of the inflammatory agent, vasodilation and redness occur because TPA mimics endogenous arachidonic acid-activating protein kinase C (PKC), which releases nuclear factor kappa B (NF-kB), starting with the overexpression of COX-2 and other proinflammatory mediators such as NO• produced by iNOS and extending its vasodilatory effect for at least 4 h. The late edema formation phase relies on COX-2 activation and prostaglandin biosynthesis, favoring plasma extravasation into the damaged tissue at 6 h and increasing ear edema diameter [54].

In a previous study on male Balb/C mice using a TPA-induced acute ear edema model, the ethanolic extract of wild-type *S. rosmarinus* leaves showed a dose-dependent effect, with 98% inhibition at 1 mg/ear, and the authors attributed this notable skin anti-inflammatory effect to its secondary metabolites, such as carnosic acid, a polyphenolic acid derivative, by selectively inhibiting COX-2 and reducing NO in a dose-dependent manner [55]; the same behavior was observed in the TPA results from the present work.

Other secondary metabolites present in the *S. rosmarinus* callus culture, such as rosmarinic acid [56], protocatechuic acid [57], and vanilloside [58], which in previous studies and individually have been shown to have various mechanisms of action, could have anti-edema effects in both murine models of acute inflammation and the vasoregulatory pathway in the TPA model: (1) they can inhibit the NF-kB pathway [59], (2) inactivate the inflammatory enzymes COX-2 and iNOS [60], (3) reduce extravasation and leukocyte infiltration, and (4) regulate the oxidative pathway of acute inflammation during the respiratory burst of phagocytes [61]. However, these possible mechanisms proposed for the observed anti-inflammatory effect are, for the moment and under this methodology, just hypotheses that require more detailed and specific direct investigation. It is important to consider that although the callus extract has some secondary metabolites and increased concentrations with respect to those of the wild-type plant extract and that it has polyphenolic compounds, which are slightly related to specific anti-inflammatory keys, the anti-inflammatory effect of the rosemary callus extract in this work should be evaluated on the same anti-inflammatory pathways as its flavonoids and compared to determine whether the increase in the polyphenolic compounds content of the callus extract is related to the increase in its anti-inflammatory effect.

One limitation of this study is the use of plant materials obtained under different conditions. The wild plant material was collected at different times and exposed to varying environmental and climatic factors, whereas the in vitro-grown material was cultivated under controlled conditions. These differences may influence the phytochemical composition and, consequently, the biological activity of the resulting extracts. Nevertheless, comparing both types of material was the main goal of this work, and the observed differences provide relevant evidence of their distinct chemical profile and biological effects. Additionally, the number of biological replicates was limited; thus, the results should be considered preliminary. Further validation using independent biological samples to more reliably assess variability between materials.

Furthermore, cell viability results obtained through the MTT assay reflect changes in cellular metabolic activity and do not constitute a direct measure of the number of viable cells. Similarly, nitrite quantification provides an indirect estimate of nitric oxide production. Accordingly, the results should be interpreted considering the inherent limitations of these methods.

Overall, the findings revealed differences among the evaluated extracts in terms of cell viability and modulation of nitrite production under the experimental conditions used. However, due to limitations associated with the origin and conditions of the biological materials, the limited number of biological replicates, the absence of extraction yield data, and the methodological constraints of the MTT assays and nitrite quantification, the findings should be interpreted as preliminary. Therefore, the conclusions of this study are limited exclusively to the extracts analyzed, the parameters assessed, and the specific experimental conditions used. Further research is needed, including independent biological samples, determination of extraction yields, and the use of complementary methodologies, such as repeated dose studies, to confirm and expand on these reported results.

## 5. Conclusions

*Salvia rosmarinus* callus culture extract resulted in higher concentrations of three major metabolites (protocatechuic acid, vanilloside, and rosmarinic acid) than did the wild-type plant extract. Both extracts exerted moderate in vitro cytotoxic effects only against cancerous cells at low concentrations and only at very high concentrations in healthy human cells. Additionally, both showed an LD_50_ > 2 g/kg after a single i.g. administration in male mice, with incipient kidney and spleen increased relative organ weight at different doses not associated with lethality for only the wild-type plant extract; in contrast, compared with the other treatments, the callus extract slightly altered the liver relative weight at intermediate and high doses.

For acute anti-inflammatory effect, the extract from the in vitro callus culture of *S. rosmarinus* exerted a moderate effect after single oral administration at a low dose, compared to wild plant extract, which showed a dose-dependent effect even greater than callus at the high tested dose. For local effect, callus extract provoked a weaker effect after single dermal application compared to the quantity-dependent manner shown by wild plant extract, but with notorious inhibition in the nitroderivative products concentration in the ear edema tissue.

The results of the present work demonstrate, in a preliminary way, how the establishment of a callus culture line of *S. rosmarinus* allowed the generation of a biomass characterized by a few bioactive secondary metabolites of pharmacological and therapeutic interest, with innovative anti-inflammatory activity and an inhibition of nitro-derived products in inflamed tissue, with no lethal effects in mice, indicating that it is a sustained and sustainable source of new possible anti-inflammatory agents.

## Figures and Tables

**Table 1:** Gradient elution profile for UPLC analysis of both *S. rosmarinus* extracts.

Time (min)	A (%)	B (%)
0	99	1
30	50	50
35	1	99
39	1	99
40	99	1
45	99	1

The flow was 0.3 mL/min. The injection volume was 2 μL. The column was an Agilent Zorbax SB-C18 column (2.1 × 50 mm, 1.8 µm), and the column temperature was 40 °C. The flow rate was 0.3 mL/min, and the sample injection volume was 2 μL. All the extracts from the callus culture and wild-type plant extract were analyzed in technical triplicate (n = 3).

**Table 2:** Mass spectrometry analysis equipment conditions for both *S. rosmarinus* extracts.

Parameter	Value
Gas Temp	300 °C
Gas Flow	5 L/min
Nebulizer	45 psi
Sheath Gas Temp	250 °C
Sheath Gas Flow	11 L/min
Capillary voltage (positive and negative)	3500 V
Nozzle voltage (positive and negative)	500 V

**Table 3:** Specific experimental conditions for MRM and mass spectrometry analysis to identify and quantify phenolic chemomarker compounds in both *S. rosmarinus* extracts.

Phenolic Chemomarker Compound	dMRM Transition			Mass Spectrometric Conditions			Quantification Conditions		
	Precursor Ion (Da)	Product Ion (Da)	Retention Time (min)	Collision Energy (V)	Fragmentor (V)	Polarity	Range (µM)	Regression Typ	R^2^
Protocatechuic acid ^1^	153.0	109.1	2.89	10	100	Negative	0.125–19	Quadratic	0.99
Vainilloside ^2^	315.1	153.0	5.04	10	100	Positive	0.125–19	Quadratic	0.99
Chlorogenic acid ^1^	355.1	163.0	6.00	10	100	Positive	0.125–19	Quadratic	0.99
4-Coumaric acid ^1^	165.0	147.0	8.16	10	100	Positive	0.125–19	Quadratic	0.99
Ferulic acid ^1^	195.1	145.0	9.63	20	100	Positive	0.125–19	Quadratic	0.99
Rosmarinic acid ^1^	361.1	163.0	13.46	10	100	Positive	0.125–19	Quadratic	0.99

The retention time variation allowed for the search for the compounds was 2 min in each case. The cell acceleration voltage was 7 V for each compound. Dilutions were made if the concentrations of some compounds were higher than the linear range. Pure compounds were purchased from the following sources: Commercially obtained from Extrasynthese ^1^ (Lyon, France) and Sigma Aldrich ^2^ (St. Louis, MO, USA).

**Table 4:** Phenolic compound contents in both extracts from *S. rosmarinus.*

No.	Identified Compound	Concentration (µg/g Extract)	
		*Sr*WE	*Sr*CE
1	Protocatechuic acid	9.82 ± 0.39 ^a^	12.42 ± 0.46 ^b^
2	Vanilloside	ND ^a^	5.63 ± 0.85 ^b^
3	Chlorogenic acid	ND ^a^	0.6 ± 0.02 *** ^a^
4	4-Coumaric acid	13.9 ± 0.21 ^a^	14.19 ± 1.64 ^a^
5	Ferulic acid	46.56 ± 0.41 ^a^	57.66 ± 7.78 ^a^
6	Rosmarinic acid	34,953.44 ± 1871.56 ^a^	77,563.09 ± 5679.21 ^b^

The data are expressed as the mean ± standard deviation (n = 3). “ND” stands for not detected. *** values below the limit of quantification. Welch’s *t* test by compound (*p* < 0.01); different superscripted letters indicate significant differences. *S. rosmarinus* wild leaf extract (*Sr*WE); *S. rosmarinus* callus extract (*Sr*CE).

**Table 5:** Records of the changes in the animal welfare parameters in male CD1 mice treated with 20, 200 and 2000 mg/Kg from *S. rosmarinus* wild extract.

Observations	30 min		4 h		24 h		48 h		1st Week		2nd Week	
	C	E	C	E	C	E	C	E	C	E	C	E
Skin and fur	N	3 × 3n, D; 4 × 2n, H	N	2 × 4n, D	N	2 × 4n, D	N	2 × 4n, D	N	1 × 5n, D	N	N
Eyes	N	N	N	N	N	N	N	N	N	N	N	N
Mucous membranes	N	N	N	N	N	N	N	N	N	N	N	N
Salivation	N	N	N	N	N	N	N	N	N	N	N	N
Lethargy	N	2 × 4n, D; A, H	N	N	N	N	N	N	N	N	N	N
Dream	N	N	N	N	N	N	N	N	N	N	N	N
Coma	N	N	N	N	N	N	N	N	N	N	N	N
Seizures	N	N	N	N	N	N	N	N	N	N	N	N
Tremors	N	2 × 4n, D; 4 × 2n, H	N	A, M	N	N	N	N	N	N	N	N
Diarrhea	N	N	N	N	N	N	N	N	N	N	N	N
Pain	N	5 × 1n, D; 3 × 3n, M; 1 × 5n, H	N	2 × 4n, D; 1 × 5n, M	N	2 × 4n, D	N	2 × 4n, D	N	1 × 5n, D	N	N
Morbidity	N	N	N	N	N	N	N	N	N	N	N	N
Mortality	N	N	N	N	N	N	N	N	N	N	N	N

C= control; E= extract; N= normal; 1 × 5n = 1 affected 5 normal; 2 × 4n = 2 affected 4 normal; 3 × 3n = 3 affected 3 normal; 4 × 2n = 4 affected 2 normal; 5 × 1n = 5 affected 1 normal; A = all affected; D = 20 mg/kg; M = 200 mg/kg; H = 2000 mg/kg.

**Table 6:** Records of the changes in the animal welfare parameters in male mice treated with 20, 200 and 2000 mg/Kg from *S. rosmarinus* callus extract.

Observations	30 min		4 h		24 h		48 h		1st Week		2nd Week	
	C	CE	C	CE	C	CE	C	CE	C	CE	C	CE
Skin and fur	N	1 × 5n, D	N	N	N	N	N	N	N	N	N	N
Eyes	N	N	N	N	N	N	N	N	N	N	N	N
Mucous membranes	N	N	N	N	N	N	N	N	N	N	N	N
Salivation	N	N	N	N	N	N	N	N	N	N	N	N
Lethargy	N	N	N	N	N	N	N	N	N	N	N	N
Dream	N	N	N	N	N	N	N	N	N	N	N	N
Coma	N	N	N	N	N	N	N	N	N	N	N	N
Seizures	N	N	N	N	N	N	N	N	N	N	N	N
Tremors	N	N	N	N	N	N	N	N	N	N	N	N
Diarrhea	N	N	N	N	N	N	N	N	N	N	N	N
Pain	N	N	N	N	N	N	N	N	N	N	N	N
Morbidity	N	N	N	N	N	N	N	N	N	N	N	N
Mortality	N	N	N	N	N	N	N	N	N	N	N	N

C = control; CE = callus extract; N = normal; 1 × 5n = 1 affected 5 normal; D = 20 mg/kg.

**Table 7:** Effects of single-administration *S. rosmarinus* callus line and wild-type plant leaf extracts on body weight and relative organ weight in healthy mice on the 14th day of treatment.

					95% Confidence Interval		
	Measurements	Treatments	N	Mean	Lower	Upper	SD
(g)	Terminal BW	Control	6	28.33333	19.73906	36.92761	8.18942
		SrWE (20 mg/kg)	6	42.00000	39.26341	44.73659	2.60768
		SrCE (20 mg/kg)	6	27.66667	22.67035	32.66298	4.76095
		SrWE (200 mg/kg)	6	42.66667	40.60307	44.73026	1.96638
		SrCE (200 mg/kg)	6	34.16667 ^a,b^	28.67674	39.65660	5.23132
		SrWE (2000 mg/kg)	6	31.00000	27.24542	34.75458	3.57771
		SrCE (2000 mg/kg)	6	33.83333 ^a,b^	30.11314	37.55353	3.54495
	Liver	Control	6	0.05927	0.05773	0.06082	0.00147
		SrWE (20 mg/kg)	6	0.07010	0.06039	0.07981	0.00925
		SrCE (20 mg/kg)	6	0.07257	0.06501	0.08012	0.00720
		SrWE (200 mg/kg)	6	0.06389	0.06115	0.06662	0.00261
		SrCE (200 mg/kg)	6	0.06682 ^a^	0.06439	0.06925	0.00231
		SrWE (2000 mg/kg)	6	0.06831	0.06359	0.07303	0.00450
		SrCE (2000 mg/kg)	6	0.07155 ^a^	0.06915	0.07394	0.00228
	Kidney	Control	6	0.01664	0.01484	0.01845	0.00172
		SrWE (20 mg/kg)	6	0.02003 ^a^	0.01811	0.02195	0.00183
		SrCE (20 mg/kg)	6	0.01664	0.01484	0.01845	0.00172
		SrWE (200 mg/kg)	6	0.01969	0.01703	0.02234	0.00253
		SrCE (200 mg/kg)	6	0.01631	0.01493	0.01768	0.00131
		SrWE (2000 mg/kg)	6	0.02165 ^a^	0.02037	0.02292	0.00122
		SrCE (2000 mg/kg)	6	0.01646 ^b^	0.01502	0.01791	0.00137
	Spleen	Control	6	0.00644	0.00540	0.00748	9.87 × 10^−4^
		SrWE (20 mg/kg)	6	0.01273 ^a^	0.00924	0.01622	0.00333
		SrCE (20 mg/kg)	6	0.00797	0.00745	0.00850	5.02 × 10^−4^
		SrWE (200 mg/kg)	6	0.01150 ^a^	0.00870	0.01430	0.00267
		SrCE (200 mg/kg)	6	0.00771	0.00580	0.00963	0.00182
		SrWE (2000 mg/kg)	6	0.01661 ^a^	0.01297	0.02024	0.00346
		SrCE (2000 mg/kg)	6	0.00815 ^a,b^	0.00673	0.00957	0.00136

Note. The CI of the mean assumes sample means follow a t-distribution with N − 1 degrees of freedom. The Shapiro-Wilk normality test and Brown–Forsythe test were used to confirm the homogeneity of variance in all the cases. Owing to the unequal variance in body weights on the 14th day post-treatment and relative liver weights at the end of the experimental design, Welch’s ANOVA and post hoc Dunnett’s T3 multiple comparison tests (*p* < 0.05) were carried out. On the other hand, owing to the equal variances in relative kidney weights of the liver and spleen at the end of the experimental time, the weights were analyzed by ordinary one-way ANOVA and Tukey’s multiple comparison test (*p* < 0.05). Superscript letters (a) indicate significant differences vs. the control mouse group, and (b) indicate significant differences vs. the S. rosmarinus wild-type extract at its own dosage level; *S. rosmarinus* wild-type leaf extract (SrWE); and *S. rosmarinus* callus extract (SrCE).

**Table 8:** Anti-inflammatory activity of the *S. rosmarinus* in vitro callus line and wild-type plant leaf extract on carrageenan-induced acute paw edema development in mice.

Treatment	Dose (mg/kg)	Paw Edema Formation (mm) at 5 h
Carrageenan control	-	0.97 ± 0.02
Indomethacin	10	0.53 ± 0.03 ^a^ (44.56%)
*Sr*CE	150	0.61 ± 0.05 ^ab^ (37.12%)
	300	0.55 ± 0.03 ^•a^ (42.94%)
	600	0.52 ± 0.05 ^•a^ (46.21%)
*Sr*WE	150	0.96 ± 0.01 ^bc^ (NE)
	300	0.70 ± 0.02 ^•abc^ (27.61%)
	600	0.40 ± 0.02 ^•^*^abc^ (58.10%)

Data are shown as the mean (±) with its SEM (n = 7), and the data in parentheses indicate the percentage of edema inhibition compared with the untreated control. Shapiro-Wilk normality test (*p* = 0.0987); two-way ANOVA, repeated measures (RM), post hoc SNK test (*p* < 0.05). Superscript letters indicate different levels of significant differences: ^•^ vs. 150 mg/kg; * vs. 300 mg/kg; ^a^ vs. Carrageenan control. ^b^ vs. Indomethacin. ^c^ vs. SrCE. *S. rosmarinus* callus extract (*Sr*CE); *S. rosmarinus* wild-type leaf extract (*Sr*WE); NE, no effect.

**Table 9:** Anti-inflammatory effects of the *S. rosmarinus* in vitro callus line and wild-type plant leaf extract on TPA-induced acute ear edema formation in mice.

Treatment.	Quantity (mg/ear)	Ear Edema Formation (mg) 6 h	Ear Edema Tissue Homogenate [nitroderivative products µM] 6 h
TPA control	-	23.90 ± 0.82	15.25 ± 0.93
Indomethacin	0.5	19.10 ± 0.18 ^a^ (19.89%)	10.69 ± 0.25 ^a^ (29.87%)
	1	15.20 ± 0.25 ^•a^ (36.40%)	11.40 ± 0.11 ^a^ (25.24%)
	2	9.20 ± 0.32 ^•*a^ (61.51%)	11.01 ± 0.28 ^a^ (27.83%)
*Sr*CE	0.5	18.70 ± 0.53 ^a^ (21.76%)	2.11 ± 0.29 ^ab^ (86.16%)
	1	18.00 ± 0.85 ^ab^ (24.69%)	0.88 ± 0.07 ^•ab^ (94.19%)
	2	17.90 ± 0.99 ^ab^ (25.10%)	0.72 ± 0.08 ^•ab^ (95.26%)
*Sr*WE	0.5	16.80 ± 0.32 ^a^ (29.71%)	6.15 ± 0.42 ^abc^ (59.67%)
	1	13.90 ± 0.77 ^•ac^ (41.84%)	6.35 ± 0.67 ^•abc^ (58.33%)
	2	14.60±0.58^•abc^ (38.91%)	4.64±0.40^•abc^ (69.57%)

Data are shown as the mean (±) with its SEM (n = 7), and the data in parentheses indicate the percentage of edema inhibition compared with that of the untreated control. Statistical analysis was performed using the Shapiro-Wilk test, two-way ANOVA, and the SNK post hoc test (*p* < 0.05); the superscripted letters indicate significant differences at different concentrations of ^•^ vs. 0.5 mg/ear. * vs. 1 mg/ear. ^a^ vs. TPA control. ^b^ vs. Indomethacin. ^c^ vs. SrCE; *S. rosmarinus* callus extract (*Sr*CE); *S. rosmarinus* wild-type leaf extract (*Sr*WE); NE, no effect.

## Data Availability

The data presented in this study are available on request from the corresponding author.

## Abbreviations

The following abbreviations are used in this manuscript:

COX-2	Induced cyclooxygenase
i.g.	Intragastrically
iNOS	Inducible nitric oxide synthase
LD_50_	Median lethal dose
NF-kB	Nuclear factor kappa-light-chain-enhancer of activated B cells
MRM	Multiple Reaction Monitoring
NO•	Nitric oxide
s.c.	Subcutaneously
ROS	Reactive oxygen species
TPA	12-O-tetradecanoyl-phorbol-13-acetate
UPLC	Ultrahigh-performance liquid chromatography
UPR	Unfolded protein response
